# Supplementary material for: Daytime Variation of Chloral Hydrate-Associated Sedation Outcomes: A Propensity-Matched Cohort Study
Source: J Clin Med. 2023 Feb 3;12(3):1245. doi: 10.3390/jcm12031245 (PMC9917952; doi:10.3390/jcm12031245)
Supplement: Supplementary file 1 [file jcm-12-01245-s001.zip › jcm-2097409-supplementary.pdf]

Supplementary Table S1. The demographic characteristics and time of day distributions in the excluded and eligible records.

	**Excluded records (n = 5638)**	**Eligible records (n = 41,831)**	***p*-Values**
Age, months, median (IQR)	14.2 (6.0, 31.3)	14.2 (4.9, 30.3)	0.19
Male, n (%)	3506 (62.2)	25081 (60.0)	**<0.01***
Weight, kg, median (IQR)	10.0 (7.0, 13.0)	10.0 (7.0, 13.0)	0.20
Type of patients, n (%)			0.84
Outpatient	3602 (63.9)	26785 (64.0)	
Inpatient	2036 (36.1)	15046(36.0)	
Procedures, n (%)			0.60
Cardiac ultrasound	1212 (21.5)	8955 (21.4)	
CT	460 (8.2)	3619 (8.7)	
Lung function	1597 (28.3)	11401 (27.3)	
MRI	1123 (19.9)	8338 (19.9)	
Hearing screen	1003 (17.8)	7691 (18.4)	
VAEP	79 (1.4)	603 (1.4)	
Others	164 (2.9)	1224 (2.9)	
Cohorts			0.93
Morning cases, n (%)	3613 (64.1)	26779 (64.0)	
Afternoon cases, n (%)	2025 (35.9)	15051 (36.0)	

Note: CT (computed tomography), MRI (magnetic resonance imaging), VAEP (visual and auditory evoked potential); * $p < 0.05$
